# Decreased risk of colorectal cancer among patients with type 2 diabetes receiving Chinese herbal medicine: a population-based cohort study

**DOI:** 10.1136/bmjdrc-2019-000732

**Published:** 2020-03-12

**Authors:** Jing-Siang Jhang, Hanoch Livneh, Shu-Yi Yang, Hui-Ju Huang, Michael W Y Chan, Ming-Chi Lu, Chia-Chou Yeh, Tzung-Yi Tsai

**Affiliations:** 1 Department of Chinese Medicine, Dalin Tzuchi Hospital, The Buddhist Tzuchi Medical Foundation, Chiayi, Taiwan; 2 Department of Biomedical Sciences, National Chung Cheng University, Chiayi, Taiwan; 3 Rehabilitation Counseling Program, Portland State University, Portland, Oregon, USA; 4 Department of Nursing, Dalin Tzuchi Hospital, The Buddhist Tzuchi Medical Foundation, Chiayi, Taiwan; 5 Epigenomics and Human Diseases Research Center, National Chung Cheng University, Chiayi, Taiwan; 6 Division of Allergy, Immunology and Rheumatology, Dalin Tzuchi Hospital, The Buddhist Tzuchi Medical Foundation, Chiayi, Taiwan; 7 School of Medicine, Tzu Chi University, Hualien, Taiwan; 8 School of Post-Baccalaureate Chinese Medicine, Tzu Chi University, Hualien, Taiwan; 9 Department of Environmental and Occupational Health, College of Medicine, National Cheng Kung University, Tainan, Taiwan; 10 Department of Nursing, Tzu Chi University of Science and Technology, Hualien, Taiwan; 11 Department of Medical Research, Dalin Tzuchi Hospital, The Buddhist Tzuchi Medical Foundation, Chiayi, Taiwan

**Keywords:** cohort, type 2 diabetes, herbal medicine, colon cancer

## Abstract

**Objectives:**

Patients with type 2 diabetes have a higher risk of colorectal cancer (CRC), but whether Chinese herbal medicines (CHMs) can reduce this risk is unknown. This study investigated the effect that CHMs have on CRC risk in patients with type 2 diabetes.

**Research design and methods:**

This cohort study used the Taiwanese National Health Insurance Research Database to identify 54 744 patients, newly diagnosed with type 2 diabetes, aged 20–70 years, who were receiving treatment between 1998 and 2007. From this sample, we randomly selected 14 940 CHMs users and 14 940 non-CHMs users, using propensity scores matching. All were followed through 2012 to record CRC incidence. Cox proportional hazards regression was used to compute the hazard ratio (HR) of CRC by CHMs use.

**Results:**

During follow-up, 235 CHMs users and 375 non-CHMs users developed CRC, incidence rates of 1.73% and 2.47% per 1000 person-years, respectively. CHM users had a significantly reduced risk of CRC compared with non-CHM users (adjusted HR=0.71; 95% CI 0.60 to 0.84). The greatest effect was in those receiving CHMs for more than 1 year. Huang-Qin, Xue-Fu-Zhu-Yu-Tang, Shu-Jing-Huo-Xue-Tang, Liu-Wei-Di-Huang-Wan, Ji-Sheng-Shen-Qi-Wan, Gan-Lu-Yin, Shao-Yao-Gan-Cao-Tang and Ban-Xia-Xie-Xin-Tang were significantly associated with lower risk of CRC.

**Conclusion:**

Integrating CHMs into the clinical management of patients with type 2 diabetes may be beneficial in reducing the risk of CRC.

**Significance of this study**

**What is already known about this subject?**

- Despite recent improvements in diabetes treatment, those with type 2 diabetes are still found to have higher risk of cancer, especially colorectal cancer (CRC).
- Recently, Chinese herbal medicines have become increasingly popular as an adjunctive treatment for patients with chronic diseases; nevertheless, association of Chinese herbal medicines and the risk of CRC among patients with type 2 diabetes is still unknown.

**What are the new findings?**

- This retrospective 15-year cohort study clarified the therapeutic effect of Chinese herbal medicines on the subsequent risk of CRC among patients with type 2 diabetes.
- The most prominent effect was observed among those receiving Chinese herbal medicines for more than 1 year.

**How might these results change the focus of research or clinical practice?**

- The reported findings serve an important role for healthcare providers in helping to guide more effective treatment strategies to improve clinical outcomes for patients with type 2 diabetes.
- The corresponding findings could serve as a reference for further pharmacological studies and clinical trials.

## Introduction

Diabetes mellitus has become an increasingly prevalent chronic condition that affects more than 400 million people worldwide.^1^ Over the past 50 years, numerous studies have linked diabetes, in particular type 2 diabetes, to a higher risk of cancer. It is noteworthy that the risk of colorectal cancer (CRC), the third most common cancer around the world, is estimated to be 27% higher in patients with type 2 diabetes than in non-diabetic control patients.^2^ According to the WHO, approximately 1.8 million patients are newly diagnosed with CRC, and over 800 000 die annually from this cancer.^3^

In Taiwan, CRC is commonly diagnosed and is the third leading cause of cancer-related death. Massive developments in specialized diagnostic and therapeutic methods have improved the survival of patients with type 2 diabetes. Some studies have indicated that the long-term use of insulin or sulfonylureas may predispose patients with type 2 diabetes to develop CRC.^4 5^ In view of this concern, it is of therapeutic interest to explore alternative treatments that may lessen this risk.

With few side effects, Chinese herbal medicines (CHMs) had been used for patients with chronic illnesses such as dementia,^6^ hepatocellular carcinoma^7^ and vertigo.^8^ Several randomized clinical trials have further suggested that CHMs could delay the progression of distal symmetric polyneuropathy^9–11^ and nephropathy.^12^ More specifically, research findings have suggested that CHMs protect against CRC risk by improving the antitumor activity of the chemotherapy drug 5-fluorouracil against the HT-29 colon cancer cell,^13^ implying that CHMs should not be neglected in the treatment of patients with type 2 diabetes.

Given the possible beneficial effect of CHMs on subsequent CRC risk, and the limited information on whether CHMs could modify the relationship between type 2 diabetes and CRC risk, findings from a long-term population-based cohort study might be useful in allocating medical resources and in instituting fact-based policymaking for the treatment of CRC among patients with type 2 diabetes. Nevertheless, to date, no clinical observations or empirical data have documented this potential benefit for patients with type 2 diabetes. Therefore, in this study, we analyzed a nationwide population-based database to compare the risk of CRC among patients with type 2 diabetes who either did or did not receive CHMs.

## Research design and methods

### Data source

For this study, we used a publicly released cohort dataset, the Longitudinal Health Insurance Database (LHID), comprised of approximately 1 000 000 randomly sampled people and obtained all records from 1996 to 2012. The database has been confirmed by the National Health Research Institute to be representative of the Taiwanese population and its data have been used in many previously published scientific papers.^14^ The encrypted information protects patient privacy and allows linkage of all claims for the same patient within the database. This database contains all National Health Insurance (NHI) enrolment files, claims data and the registry for prescription drugs and provides comprehensive information on all individuals covered by the insurance program. This study was conducted in accordance with the Helsinki Declaration and was evaluated and approved by the local Institutional Review Board and ethics committee of Buddhist Dalin Tzu Chi Hospital, Taiwan (No. B10004021-2).

### Study population

**Figure 1:**
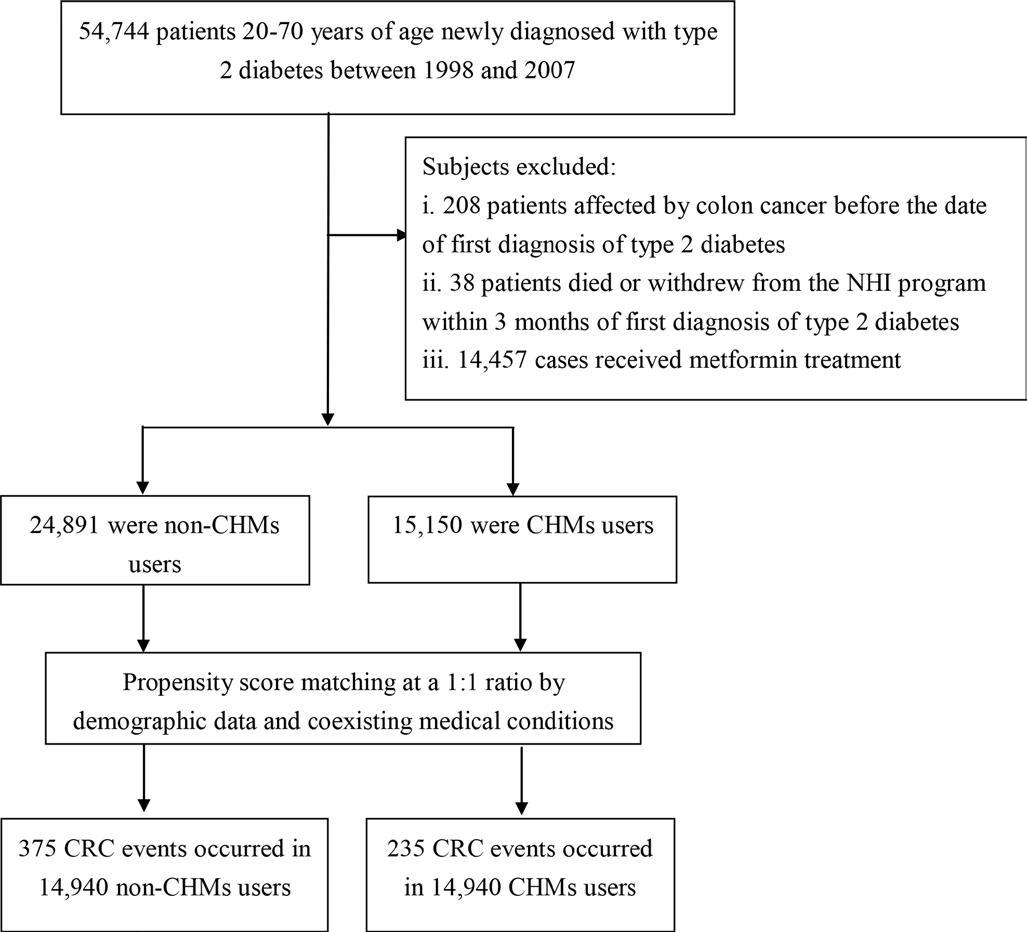
Flowchart of study and follow-up of study participants. CHMs, Chinese herbal medicines; NHI, National Health Insurance.

Patients, 20–70 years of age, newly diagnosed with type 2 diabetes between 1998 and 2007 were identified (figure 1). To be included, patients with type 2 diabetes had to have at least three ambulatory or inpatient claims with the International Classification of Disease, 9th edition, Clinical Modification (ICD-9-CM) diagnosis code 250 and excluding type 1 diabetes (ICD-9-CM code 2501). A total of 208 cases with type 2 diabetes were then excluded because of prior diagnosis of CRC (ICD-9-CM codes colon cancer: 153x, rectal cancer: 154x). This exclusion was indicated by linking type 2 diabetes subjects to the catastrophic illness registry. In Taiwan, insured residents with major diseases (eg, cancer, autoimmune diseases, chronic renal failure) can apply for a catastrophic illness certificate that grants exemption from copayment. Those with a follow-up period <3 months were also excluded (n=38). Additionally, given the protective effect of metformin in decreasing the onset of cancer,^15^ those patients who received in the past metformin with an average dose of more than 250 mg per day were also excluded (n=14 457). Overall, we identified 40 041 subjects with new-onset type 2 diabetes.

In Taiwan, only certified Chinese medicine physicians are entitled to prescribe CHMs. We used the frequency of visits to a Chinese medicine physician to verify the CHMs exposure of each subject with type 2 diabetes. Those receiving CHMs after their initial diagnosis of type 2 diabetes for more than 30 days were identified as CHMs users, whereas those treated for 30 days or less were considered non-CHMs users.^8^ Based on this rule, 15 150 cases were designated as CHMs users. A comparison cohort was randomly selected from the remaining insured type 2 diabetes cases without CHMs use. For each type 2 diabetes case who received CHMs treatment, one control case without CHMs treatment was selected, using a 1:1 match based on a propensity score. Propensity scores representing the likelihood of receiving CHMs were calculated using logistic regression analysis, conditional on the baseline covariates listed in table 1. Accordingly, an equal number of patients in the CHMs and non-CHMs cohorts were analyzed in this study. The index date for the follow-up period for non-CHMs users was the date of the type 2 diabetes diagnosis; the index date for the follow-up period for CHMs users was the first date of the initiation of CHMs service. The end date of the follow-up period for both groups was the earliest of the following: (1) having a diagnosis of CRC; (2) withdrawing from the insurance program or (3) the date of December 31, 2012.

**Table 1:** Demographic data and comorbidity comparison of the study subjects

Variables	CHMs non-users	CHMs users	P value
	n=14 940 (%)	n=14 940 (%)	
Age, (years)			0.75
≤50	5259 (35.2)	5274 (35.3)	
>50	9681 (64.8)	9666 (64.7)	
Mean (SD)	53.93±10.32	53.86±10.42	0.74
Gender			0.50
Female	8396 (56.2)	8337 (55.8)	
Male	6544 (43.8)	6603 (44.2)	
Monthly income			0.51
Low	5692 (38.1)	5677 (38.0)	
Median	8411 (56.3)	8381 (56.1)	
High	837 (5.6)	882 (5.8)	
Residential area			0.28
Urban	8725 (58.4)	8650 (57.9)	
Suburban	2256 (15.1)	2346 (15.7)	
Rural	3959 (26.5)	3944 (26.5)	
Comorbidity			
Hypertension	6917 (46.3)	6872 (46.0)	0.49
Stroke	1121 (7.5)	1150 (7.7)	0.53
Heart disease	2928 (19.6)	2928 (19.6)	0.99
Chronic kidney disease	194 (1.3)	194 (1.3)	0.67
Depression	493 (3.3)	538 (3.6)	0.16
Rheumatologic disorders	105 (0.7)	105 (0.7)	0.77

CHMs, Chinese herbal medicines.

### Covariate assessment

Covariates comprised the baseline sociodemographic characteristics and comorbidities. Sociodemographic data included patient’s age, sex, income (for estimating insurance payments) and urbanization level of the residential area. Monthly income was stratified into New Taiwan Dollar (NTD) ≤17 880; NTD 17 881–NTD 43 900 and ≥NTD 43 901. Urbanization levels were divided into urban (levels 1–2), suburban (levels 3–4) and rural (levels 5–7). Level 1 refers to the “most urbanized” communities and level 7 refers to the “least urbanized”.^16^ Baseline comorbidities included hypertension (ICD-9-CM 401–405), stroke (ICD-9-CM 430–438), heart disease (ICD-9-CM 410–429), chronic kidney disease (ICD-9-CM 585), depression (ICD-9-CM 296.2, 296.3, 300.4 and 311) and rheumatologic disorders (ICD-9-CM 725–729); all were based on each individual’s medical records for the 1 year prior to initial cohort entry.

### Statistical analysis

We conducted χ^2^ test and *t*-test analyses to examine the differences in demographic characteristics and comorbidities between patients with type 2 diabetes with and without CHMs treatment. The incidence rate of CRC was calculated as the number of cases per 1000 person-years (PYs). Multivariate Cox proportional hazards regression analysis was then applied to compute the HR with 95% CI of CRC risk in association with CHMs use. To test the robustness of the relationship between CHMs use and subsequent CRC risk, we divided the CHMs users into two subgroups: those who used CHMs for 30–365 days and those who used CHMs for more than 365 days. Analysis stratified by age and sex using Cox proportional hazards regression was also conducted to assess the HR of CRC among those who did and did not receive CHMs. Assumptions of the proportional hazards model were verified by using plots of log (-log (survival)) function) versus log (time) and Schoenfeld residuals versus time. All analyses were conducted using SAS V.9.3 software (SAS Institute, Cary, North Carolina, USA), at a p<0.05 statistically significant level.

## Results

The CHMs user and non-CHMs user cohorts provided, each, data for 14 940 subjects. After the matching procedure with propensity score, no significant differences were observed between the two groups on age, sex, monthly income, residential area and comorbidities, indicating that the two groups were comparable with regard to these variables (table 1).

Among all eligible subjects, 610 first episodes of CRC occurred, 375 in non-CHMs users and 235 in CHMs users, during follow-up periods of 151 745.04 and 135,251.86 PYs, respectively. The incidence rate of CRC was significantly lower in CHMs users than in non-CHMs users (1.73 vs 2.47, respectively, per 1000 PYs), with an adjusted HR of 0.71 (95% CI 0.60 to 0.84) (table 2). Of note, those who used CHMs for more than 365 days had a lower risk of CRC (95% CI 0.28 to 0.61) vs non-users by 59%.

**Table 2:** Crude and adjusted HR of colorectal cancer for patients with type 2 diabetes with and without CHMs treatment

Patient group	Event	PYs	Incidence	Crude HR (95% CI)	Adjusted HR* (95% CI)
CHMs non-users	375	151 745.04	2.47	1.00	1
CHMs users	235	135 251.86	1.73	0.70 (0.60 to 0.83)	0.71 (0.60 to 0.84)
CHMs use for 31–365 days	208	108 166.55	1.92	0.78 (0.66 to 0.93)	0.79 (0.67 to 0.94)
CHMs use >1 year	27	27 085.31	1.00	0.40 (0.27 to 0.60)	0.41 (0.28 to 0.61)

Incidence rate is per 1000 PYs.

*Model adjusted for age, gender, urbanization level, monthly income and comorbidity.

CHMs, Chinese herbal medicines; PYs, patient-years.

Table 3 presents the results from this analysis stratified by age and gender. Collectively, a more significant beneficial effect of CHMs was observed among younger subjects, irrespective of sex. Furthermore, the multivariable stratified analysis verified that the benefit of CHMs therapy for treating CRC was more predominant in males than in females, with an adjusted HR of 0.66 and 0.76, respectively (table 3). The 10 most commonly prescribed herbal formulae for those with type 2 diabetes are summarized in table 4. Among them, one was a single-herb product while the remaining products were multiple-herb products. Cox proportional hazards regression analysis showed that the use of Huang-Qin, Shu-Jing-Huo-Xue-Tang, Xue-Fu-Zhu-Yu-Tang, Ji-Sheng-Shen-Qi-Wan, Liu-Wei-Di-Huang-Wan, Gan-Lu-Yin, Shao-Yao-Gan-Cao-Tang and Ban-Xia-Xie-Xin-Tang was significantly associated with lower risk of CRC (table 4).

**Table 3:** Age-specific and sex-specific incidence and adjusted HR of colorectal cancer in relation to CHMs use in patients with type 2 diabetes

Variables	CHMs non-users			CHMs users			Crude HR (95% CI)	Adjusted HR (95% CI)
	Case	PYs	Incidence	Case	PYs	Incidence		
Female								
≤50 years	33	26 100.8	1.26	17	26 277.02	0.65	0.50 (0.28 to 0.90)	0.50 (0.27 to 0.89)*
>50 years	154	58 674.38	2.62	107	48 944.48	2.19	0.84 (0.66 to 1.07)	0.84 (0.65 to 1.07)*
All	187	84 775.18	2.21	124	75 221.5	1.65	0.76 (0.59 to 0.94)	0.76 (0.61 to 0.96)†
Male								
≤50 years	39	25 323.32	1.54	20	22 241.56	0.90	0.60 (0.35 to 0.98)	0.59 (0.34 to 0.97)*
>50 years	149	41 646.54	3.58	91	37 788.8	2.41	0.67 (0.52 to 0.87)	0.68 (0.52 to 0.88)*
All	188	66 969.86	2.81	111	60 030.36	1.85	0.65 (0.52 to 0.83)	0.66 (0.53 to 0.84)†

Incidence rate is per 1000 PYs.

*Model adjusted for urbanization level, monthly income and comorbidity.

†Model adjusted for age, urbanization level, monthly income and comorbidity.

CHMs, Chinese herbal medicines; PYs, person-years.

**Table 4:** Risk of colorectal cancer in relation to the 10 most commonly used CHMs for patients with type 2 diabetes

CHMs name	Ingredients or generic name	Frequency of prescriptions	Crude HR (95% CI)	Adjusted HR * (95% CI)
Single-herb products				
Huang-Qin	*Scutellariae radix*	15 796	0.67 (0.52 to 0.84)	0.68 (0.56 to 0.90)
Multiherb products				
Shu-Jing-Huo-Xue-Tang	*Paeoniae radix, Angelicae radix, Atractylodis lanceae rhizome, Cnidii rhizome, Persicae semen, Poria, Rehmanniae radix, Achyranthis radix, Clematis radix, Gentianae scabrae radix, Notoptergii rhizome, Saposhnikoviae radix, Sinomeni caulis et rhizome, Angelicae dahuricae radix, Aurantii nobillis pericarpium, Glycyrrhizae radix, Zingiberis rhizoma*	18 503	0.65 (0.54 to 0.78)	0.69 (0.56 to 0.81)
Xue-Fu-Zhu-Yu-Tang	*Glycyrrhizae radix, Angelicae sinensis radix, Rehmanniae radix, Achyranthis bidentatae radix, Persicae semen, Chuanxiong rhizoma, Platycodonis radix, Carthami flos, Paeoniae radix rubra, Bupleuri radix, Aurantii fructus*	11 664	0.56 (0.44 to 0.72)	0.60 (0.46 to 0.76)
Ji-Sheng-Shen-Qi-Wan	*Rehmanniae radix preparata, Fructus corni, Cortex moutan, Rhizoma dioscoreae, Poria and Rhizoma alismatis, Achyranthis bidentatae radix, Aconiti lateralis praeparata radix, Cinnamomi cortex, Plantaginis semen*	12 498	0.55 (0.42 to 0.72)	0.53 (0.40 to 0.69)
Liu-Wei-Di-Huang-Wan	*Rehmanniae radix preparata, Fructus corni, Cortex moutan, Rhizoma dioscoreae, Poria, Rhizoma alismatis*	11 031	0.58 (0.49 to 0.67)	0.62 (0.53 to 0.72)
Gan-Lu-Yin	*Glycyrrhilza uralensis, Liriope spicata, Citrus sinensis, Rehmannia glutinose, Artemisia capillaris, Eriobotrya japonica, Dendrobium nobile, Scutellaria baicalensis and Asparagus cochinchinensis*	9753	0.62 (0.47 to 0.81)	0.64 (0.49 to 0.84)
Du-Huo-Ji-Sheng Tang	*Angelicae pubescentis radix, Taxilli herba, Eucommiae cortex, Cyathulae radix, Asari radix, Gentianae macrophyllae radix, Poria, Cinnamomi cortex, Saposhnikoviae radix, Chuanxiong rhizoma, Ginseng radix, Glycyrrhizae radix, Angelicae sinensis radix, Paeoniae radix alba, Rehmanniae radix*	9063	0.79 (0.64 to 0.97)	0.76 (0.61 to 1.01)
Shao-Yao-Gan-Cao-Tang	*Paeoniae radix, Glycyrrhizae radix*	8003	0.74 (0.60 to 0.91)	0.75 (0.57 to 0.89)
Yu-Quan-Wan	*Ophiopogonis radix, Ginseng radix, Poria, Astragali radix, Mume fructus, Trichosanthis fructus, Glycyrrhizae radix, Puerariae lobatae radix*	4932	0.88 (0.56 to 1.13)	0.92 (0.60 to 1.23)
Ban-Xia-Xie-Xin-Tang	*Pinelliae rhizoma, Scutellariae radix, Zingiberis rhizoma, Ginseng radix, Glycyrrhizae radix, Coptidis rhizoma, Jujubae fructus*	4529	0.69 (0.52 to 0.91)	0.70 (0.54 to 0.97)

*Model adjusted for age, gender, urbanization level, monthly income and comorbidities.

CHMs, Chinese herbal medicines.

## Discussion

This is the first evidence-based cohort study addressing the association between CHMs use and CRC risk in patients with type 2 diabetes through a large nationwide claims-based data source. In this follow-up study of 15 years (1998–2012), we found that patients with type 2 diabetes who were receiving CHMs had a nearly 30% lower chance of CRC than those not using CHMs. Furthermore, those receiving CHMs for more than 365 days were found to have a lower risk of CRC by nearly 60%. The dose-response relationship may elucidate the causality between CHMs use and the decrease in the predisposition for developing CRC. No previous studies have been conducted to determine the long-tern impact of CHMs on CRC risk among patients with type 2 diabetes, thus rendering a comparison of results impossible. However, the findings obtained herein are consistent with earlier research findings and add to the growing body of knowledge on the beneficial effects of CHMs for the patients with chronic diseases.^6–8^

The effect of CHMs was further analyzed with stratification by age and sex. Taken together, the use of CHMs was found to have a greater effect on CRC risk in younger patients irrespective of their sex, and th finding echod these of previous studies.^7 8^ We speculated that younger patients may have fewer coexisting medical conditions or possess better medical knowledge and coping resources, in addition to demonstrating more positive attitudes toward medical impairments,^17^ thus enhancing the preventive impact of CHMs on CRC risk.

An additional contribution of the present study is the list of Chinese herbal products that were found to be beneficial in reducing CRC risk. For example, Huang-Qin is one of the most common CHMs used to treat type 2 diabetes and it may help in lessening the subsequent risk for CRC. Wogonin, the main ingredient in Huang-Qin, was found to have antineoplastic and anti-inflammatory effects in both in vitro and in vivo studies. One study found that wogonin can induce activation of AMP-activated protein kinase (AMPK), to inhibit the proliferation and induce apoptosis in cancer cells.^18^ Additionally, other researchers have proposed that wogonin may suppress the production of interleukin (IL)-6 and IL-1b by modulating the nuclear factor (NF)-kappa beta and NF-E2-related factor 2 signaling pathways.^19^ IL-6 and IL-1β are well known pleiotropic proinﬂammatory cytokines with profound effects on several diseases, especially CRC onset.^20 21^

Findings of the present study revealed that the other commonly-prescribed formulas targeting type 2 diabetes, such as Xue-Fu-Zhu-Yu-Tang and Shu-Jing-Huo-Xue-Tang, also significantly lowered CRC risk. Recently, both animal experiments and human studies showed that Xue-Fu-Zhu-Yu-Tang has anti-inflammatory and anti-tumor effects, primarily via inhibiting the activation of such intracellular signaling pathways as phosphoinositide 3-kinase-protein kinase B-mammalian target of rapamycin (PI3K-AKT-mTOR).^22 23^ Blocking PI3K-AKT-mTOR signaling may impact the expression levels of elements in the tumor microenvironment, affecting cancer stem cell expression and survival.^24^ In addition, the positive therapeutic effect of Shu-Jing-Huo-Xue-Tang on subsequent risk of CRC may be inferred from its reported effect of increasing blood circulation and enhancing antioxidant enzymatic activity,^25^ in addition to its anti-inflammatory activity.^26^ All of these processes have been implicated in the development of colitis-associated neoplasia.^27^

Similar to a previous report,^28^ the current study showed a preventive effect of Liu-Wei-Di-Huang-Wan on the risk of CRC. This herbal product may induce the inactivation of the IGF pathway. IGF is well known to promote malignant transformation, promoting cell proliferation and dedifferentiation and inhibiting apoptosis, which in turn predisposes the individual to developing cancer.^29^ Ji-Sheng-Shen-Qi-Wan, also known as Gosha-jinki-gan, is widely used to treat patients with diabetic neuropathy. This medication enhances nitric oxide (NO) production and thereby increases blood circulation while inhibiting blood coagulation.^30^ Recently, NO has been suggested to modulate different cancer-related events, including angiogenesis, apoptosis and metastasis,^31^ all possible mechanisms for the positive effect observed here.

Gan-Lu-Yin was also found to decrease the risk of CRC. In one study, relative to untreated controls, Gan-Lu-Yin-fed rats had markedly reduced cell proliferation and migration, through the induced differentiation of WEHI-3 cells.^32^ This inhibition of angiogenesis may prevent subsequent tumor growth. Use of another common CHMs, Shao-Yao-Gan-Cao-Tang, was also related to a lower risk of CRC. This compound may rescue the decreased phosphorylation of glycogen synthase kinase 3 (GSK-3) in patients with type 2 diabetes.^33^ Activation of GSK-3 would then downregulate the PI3K-AKT-mTOR signaling pathway to inhibit colon cancer cell proliferation.^34^

Use of Ban-Xia-Xie-Xin-Tang was found to lessen the risk of developing CRC in patients with type 2 diabetes. One possible mechanism may involve the anti-inflammatory properties of Ban-Xia-Xie-Xin-Tang,^35 36^ which is often used to treat various digestive inflammations, like colitis, esophagitis and gastritis.^37^ Chronic inflammation of the bowel has been hypothesized as the most important mechanism driving CRC onset.^38^

While our study is the first to investigate CHMs effect on reducing CRC risk among patients with type 2 diabetes, there are important limitations to consider. First, a coding error may occur in the retrospective study design due to factors related to the availability and accuracy of the medical record. Therefore, we enrolled patients with type 2 diabetes and CRC only after these patients had at least three outpatient visits reporting consistent diagnoses, or after the patients had at least one inpatient admission. The CRC cases were further verified using the catastrophic illness registry. It should also be noted that the Taiwan NHI randomly audits hospital claims, interviews patients and reviews medical charts to verify the accuracy of medical records. Second, the LHID does not include detailed information on the risk factors associated with CRC, such as smoking, alcohol consumption, level of obesity or dietary habits. Future research, considering these untested variables, is needed to better assess whether the present findings are replicable across diverse groups of individuals. Third, prescriptions for medications issued before 1996 were not reflected in our data analysis. This omission may have resulted in underestimation of the cumulative frequency of prescriptions and therefore may have weakened the apparent effects of the specified herbal products. Although several commonly-prescribed CHMs for patients with type 2 diabetes were identified as influential in decreasing the risk of CRC, their therapeutic effect and safety concerns remain to be elucidated in future pharmacological investigations. Fourth, we conducted two sensitivity analysis to confirm the relationships of interest. First, we did not exclude those receiving metformin with an average dose of more than 250 mg per day (n=14 457). The reanalysis showed that the protective effect of CHMs was somewhat attenuated, but was still statistically significant (adjusted HR=0.76; 95% CI 0.64 to 0.89). Second, all CRC cases were divided into two groups based on the location of CRC, namely, the occurrence of malignant neoplasm of colon and rectal cancer. Of the 610 CRC cases, 395 were of colon cancer and 215 cases were attributed to rectal cancer. After adjusting for the confounders, the reanalysis indicated that integrating CHMs into the conventional therapy decreased the risk of colon cancer and rectal cancer, with an adjusted HR of 0.77 (95% CI 0.64 to 0.95) and 0.60 (95% CI 0.45 to 0.79), respectively. Taken together, the results from the reanalysis of the two sensitivity analyses lent support to the earlier findings of this study. Fifth, although our study revealed a substantial benefit resulting from the use of CHMs among patients with type 2 diabetes, it must be recognized that in our study patients were not randomly categorized into users and nonusers of CHMs. Therefore, potential biases may still remain, and in particular when they stem from unmeasured or unknown confounders. Caution, therefore, must be exercised when interpreting these findings.

These limitations notwithstanding, this study also offer several strengths. These include the immediate availability of data, the comprehensiveness of the database, and the statistical power derived from the large sample. In addition, a long observational period offers the opportunity to determine in detail the association between CHMs usage and CRC risk among patients with type 2 diabetes, and the available findings may serve as a useful reference for future studies focusing on additional clinical outcomes, such as colorectal neoplasm.

## Conclusion

In summary, this large-scale nationwide cohort study demonstrates that the integration of CHMs, during treatment for type 2 diabetes, appears to lower the subsequent risk of CRC by nearly 30%. We believe that these findings could serve as a reference for healthcare providers in helping to establish more effective therapeutic interventions to improve the prognosis of patients with type 2 diabetes.
